# Supplementary material for: Multiomic analysis of Schistosoma mansoni reveals unique expression profiles in cercarial heads and tails
Source: Commun Biol. 2021 Jul 12;4:860. doi: 10.1038/s42003-021-02366-w (PMC8275615; doi:10.1038/s42003-021-02366-w)
Supplement: Supplementary file 2 — Supplementary Information [file 42003_2021_2366_MOESM2_ESM.pdf]

Supplementary Information

Supplementary Data 1 contains supporting data for Figures 1, 2, 6, and Supplementary Figure 2. Supplementary Data 2 contains supporting data for Supplementary Figure 1. Supplementary Data 3 contains supporting data for Figures 3 and 4. Supplementary Data 4 contains supporting data for Figure 5.

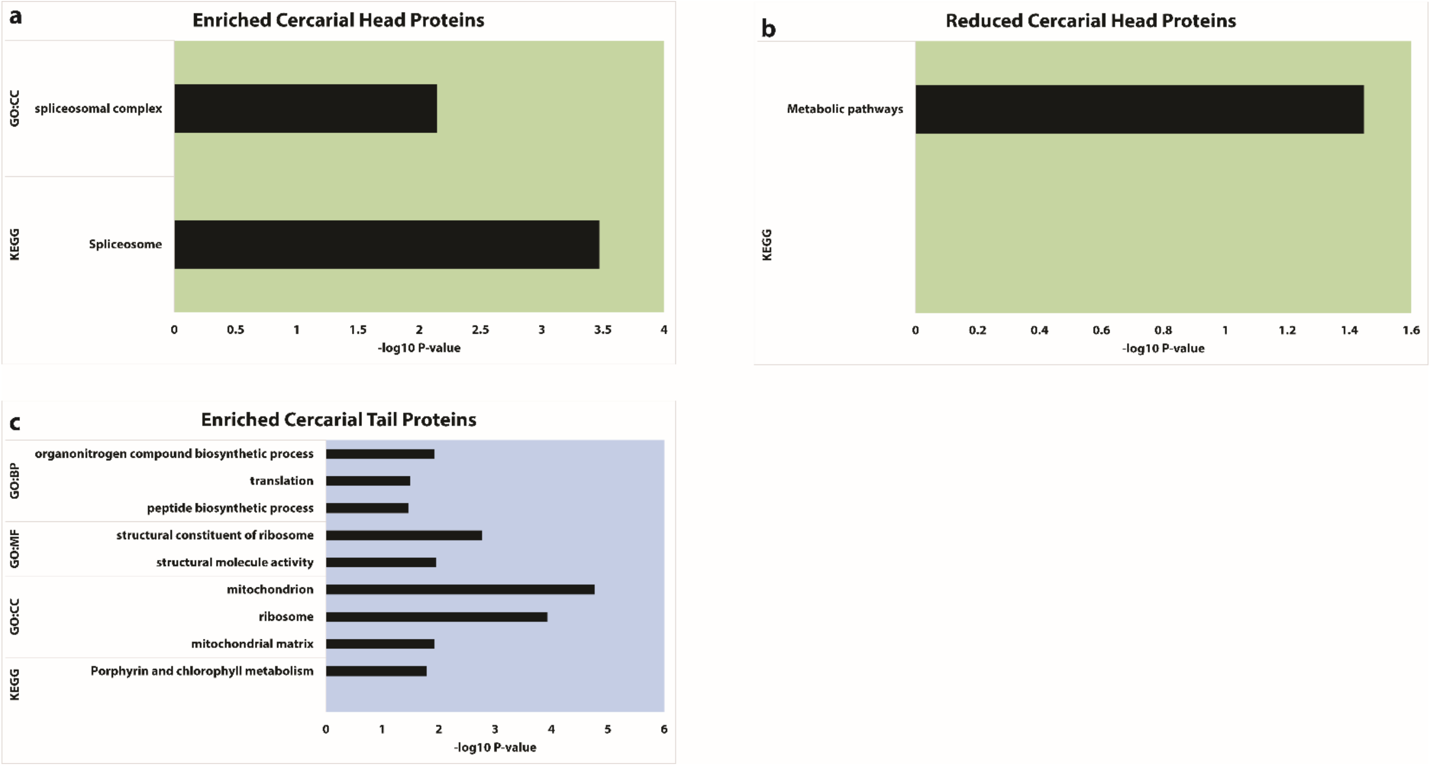

Supplementary Fig. 1. GO analysis of unique proteins from cercarial heads and tails. Panel A shows enriched proteins in cercarial heads. Panel B and Panel C are enriched and reduced unique proteins from cercarial tails, respectively. No reduced protein groups were found for cercarial heads. GO terms are shown across biological process (BP), molecular function (MF), and cell compartment (CC). All supporting data are contained in Supplementary Data 2.

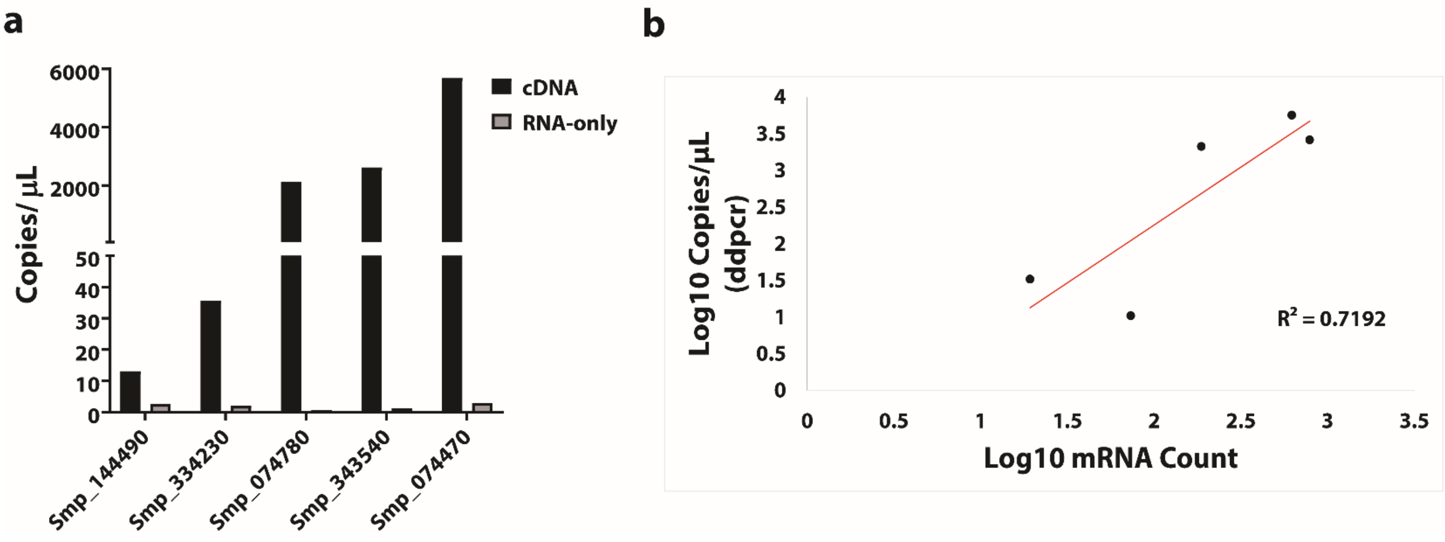

Supplementary Fig. 2 RNA-Seq mRNA abundance was verified by digital droplet PCR in cercarial tails. Panel A shows copies/µL of 5 representative genes. cDNA amplification is shown with black bars and RNA-only negative control amplification is shown with grey bars. Digital Droplet PCR was performed to acquire absolute quantitation of each gene. Analysis was performed in duplicate with RNA-only negative control was performed in duplicate as well, n = 2 independent biological replicates with > 10,000 technical replicates each. Panel B shows Log10 copies/µL along the X-axis and Log10 normalized mRNA count along the Y-axis. R2=0.7192 with red linear fit line and Spearman ranked correlation is 0.8. All supporting data are contained in Supplementary Data 1.
