# Supplementary material for: Multiomic analysis of Schistosoma mansoni reveals unique expression profiles in cercarial heads and tails
Source: Commun Biol. 2021 Jul 12;4:860. doi: 10.1038/s42003-021-02366-w (PMC8275615; doi:10.1038/s42003-021-02366-w)
Supplement: Supplementary file 3 — Description of Additional Supplementary Files [file 42003_2021_2366_MOESM3_ESM.pdf]

Description of Additional Supplementary Files

File name: Supplementary Data 1
Description: Full list of identification, differential expression, and abundance of transcripts and proteins.

File name: Supplementary Data 2
Description: Full GO enrichment term lists for unique proteins.

Title: Supplementary Data 3
Description: Full GO enrichment term lists for differential expression transcripts and proteins.

File name: Supplementary Data 4
Description: Full GO enrichment term list for differentially expressed protein re-analysis from Sotillo et al. data set. List of over-expressed proteins in 3-hour, 2-day, and 5-day schistosomula. List of protein identifications verified by the identification of Sotillo et al. 2015.
