# Supplementary material for: Multiomic analysis of Schistosoma mansoni reveals unique expression profiles in cercarial heads and tails
Source: Commun Biol. 2021 Jul 12;4:860. doi: 10.1038/s42003-021-02366-w (PMC8275615; doi:10.1038/s42003-021-02366-w)
Supplement: Supplementary file 8 — Reporting Summary [file 42003_2021_2366_MOESM8_ESM.pdf]

nature research

Corresponding author(s): Emmitt R. Jolly

Last updated by author(s): Jun 3, 2021

Reporting Summary

Nature Research wishes to improve the reproducibility of the work that we publish. This form provides structure for consistency and transparency in reporting. For further information on Nature Research policies, see our Editorial Policies and the Editorial Policy Checklist.

Please do not complete any field with "not applicable" or n/a. Refer to the help text for what text to use if an item is not relevant to your study.
For final submission: please carefully check your responses for accuracy; you will not be able to make changes later.

Statistics

For all statistical analyses, confirm that the following items are present in the figure legend, table legend, main text, or Methods section.

n/a	Confirmed	
☐	☒	The exact sample size (n) for each experimental group/condition, given as a discrete number and unit of measurement
☐	☒	A statement on whether measurements were taken from distinct samples or whether the same sample was measured repeatedly
☐	☒	The statistical test(s) used AND whether they are one- or two-sided<br>*Only common tests should be described solely by name; describe more complex techniques in the Methods section.*
☒	☐	A description of all covariates tested
☐	☒	A description of any assumptions or corrections, such as tests of normality and adjustment for multiple comparisons
☐	☒	A full description of the statistical parameters including central tendency (e.g. means) or other basic estimates (e.g. regression coefficient) AND variation (e.g. standard deviation) or associated estimates of uncertainty (e.g. confidence intervals)
☐	☒	For null hypothesis testing, the test statistic (e.g. F, t, r) with confidence intervals, effect sizes, degrees of freedom and P value noted<br>*Give P values as exact values whenever suitable.*
☒	☐	For Bayesian analysis, information on the choice of priors and Markov chain Monte Carlo settings
☒	☐	For hierarchical and complex designs, identification of the appropriate level for tests and full reporting of outcomes
☐	☒	Estimates of effect sizes (e.g. Cohen's d, Pearson's r), indicating how they were calculated

Our web collection on statistics for biologists contains articles on many of the points above.

Software and code

Policy information about availability of computer code

Data collection	Manufacturer's (ThermoScientific) software for Thermo Orbitrap Mass Spectrometers was used to produce raw mass spectrometry output files.<br>Manufacturer's (Illumina) software for the HiSeq 2500 instrument was used to collect RNA-seq data.<br>Manufacturer's (Biorad) software for QX200 ddpcr instrument was used to collect ddPCR data.
Data analysis	Transcriptome analysis was performed using FastQC (open source), Trimmomatic (open source), Salmon (open source), DEseq2 (open source), and riboPicker (open source). Transcripts were mapped to the Schistosoma mansoni genome version 14 available from (https://parasite.wormbase.org/), the SILVA small rRNA data (large subunit version 132) (small subunit version 138), and Rfam was used for 5S and 5.8S subunit rRNA sequences (release 14.2).<br><br>Proteomic analiysis was performed using PEAKSX+(Bioinformatics Solutions Inc.), Scaffold PerSPECtive (version 3.1.0, Proteome Software Inc.), Proteins were mapped to the Schistosoma mansoni genome version 14 available from (https://parasite.wormbase.org/) and the common Repository of Adventitious Proteins (cRAP v2012.01.01) sequences.<br><br>GO analysis was performed using gProfiler webtool (https://biit.cs.ut.ee/gprofiler/gost).<br>ddPCR was analyzed using ddPCRquant webtool (https://ddpcrquant.ugent.be).

For manuscripts utilizing custom algorithms or software that are central to the research but not yet described in published literature, software must be made available to editors and reviewers. We strongly encourage code deposition in a community repository (e.g. GitHub). See the Nature Research guidelines for submitting code & software for further information.

Data

Policy information about availability of data

All manuscripts must include a data availability statement. This statement should provide the following information, where applicable:

- Accession codes, unique identifiers, or web links for publicly available datasets
- A list of figures that have associated raw data
- A description of any restrictions on data availability

All data supporting the findings of this paper are contained within the paper or with the provided Supplementary Information and Supplementary Data files. A summary of source data for each figure is listed in the figure legends and the Supplementary Information file. The mass spectrometry proteomics data have been deposited to the ProteomeXchange Consortium via the PRIDE partner repository with the dataset identifier PXD026435 and DOI: 10.6019/PXD026435. The RNA-Seq data analyzed in this study are available on the National Center for Biotechnology Information (NCBI) database under the BioProject PRJNA734345.

Field-specific reporting

Please select the one below that is the best fit for your research. If you are not sure, read the appropriate sections before making your selection.

☒ Life sciences ☐ Behavioural & social sciences ☐ Ecological, evolutionary & environmental sciences

For a reference copy of the document with all sections, see nature.com/documents/nr-reporting-summary-flat.pdf

Life sciences study design

All studies must disclose on these points even when the disclosure is negative.

Sample size	Sample sizes were chosen to enable robust and unambiguous mapping of peptides/sequencing reads based upon our previous work in this area, as well as what is typical in the literature.<br>Proteomics: three biological replicates for each parasite macroscstructure<br>Transcriptomics: three biological replicates for each parasite macrostructure<br>ddPCR: Performed with 2 biological replicates and ~15,000 technical replicates per sample
Data exclusions	No data were excluded.
Replication	Transcriptomics were conducted in biological triplicate. Proteomics were conducted in biological triplicate. ddPCR was performed in biological duplicate.
Randomization	No randomization was performed,
Blinding	No blinding was performed.

Reporting for specific materials, systems and methods

We require information from authors about some types of materials, experimental systems and methods used in many studies. Here, indicate whether each material, system or method listed is relevant to your study. If you are not sure if a list item applies to your research, read the appropriate section before selecting a response.

Materials & experimental systems

n/a	Involved in the study	
☒	☐	Antibodies
☒	☐	Eukaryotic cell lines
☒	☐	Palaeontology and archaeology
☒	☐	Animals and other organisms
☒	☐	Human research participants
☒	☐	Clinical data
☒	☐	Dual use research of concern

Methods

n/a	Involved in the study	
☒	☐	ChIP-seq
☒	☐	Flow cytometry
☒	☐	MRI-based neuroimaging

This checklist template is licensed under a Creative Commons Attribution 4.0 International License, which permits use, sharing, adaptation, distribution and reproduction in any medium or format, as long as you give appropriate credit to the original author(s) and the source, provide a link to the Creative Commons license, and indicate if changes were made. The images or other third party material in this article are included in the article's Creative Commons license, unless indicated otherwise in a credit line to the material. If material is not included in the article's Creative Commons license and your intended use is not permitted by statutory regulation or exceeds the permitted use, you will need to obtain permission directly from the copyright holder. To view a copy of this license, visit http://creativecommons.org/licenses/by/4.0/
